# mRNA Transcript Variants Expressed in Mammalian Cells

**DOI:** 10.3390/ijms26031052

**Published:** 2025-01-26

**Authors:** Yashica Sharma, Kevin Vo, Sharmin Shila, Anohita Paul, Vinesh Dahiya, Patrick E. Fields, M. A. Karim Rumi

**Affiliations:** Department of Pathology and Laboratory Medicine, University of Kansas Medical Center, Kansas City, KS 66160, USA; yashica2025@gmail.com (Y.S.); kvo5@kumc.edu (K.V.); sharminshila.mib@gmail.com (S.S.); apaul04@g.ucla.edu (A.P.); vineshdahiyampharm@gmail.com (V.D.); pfields@kumc.edu (P.E.F.)

**Keywords:** transcription start sites, post-transcriptional processing of mRNAs, alternative splicing, alternative polyadenylation sites, RNA modifications

## Abstract

Gene expression or gene regulation studies often assume one gene expresses one mRNA. However, contrary to the conventional idea, a single gene in mammalian cells can express multiple transcript variants translated into several different proteins. The transcript variants are generated through transcription from alternative start sites and alternative post-transcriptional processing of the precursor mRNA (pre-mRNA). In addition, gene mutations and RNA editing further enhance the diversity of the transcript variants. The transcript variants can encode proteins with various domains, expanding the functional repertoire of a single gene. Some transcript variants may not encode proteins but function as non-coding RNAs and regulate gene expression. The expression level of the transcript variants may vary between cell types or within the same cells under different biological conditions. Transcript variants are characteristic of cell differentiation in a particular tissue, and the variants may play a key role in normal development and aging. Studies also reported that some transcript variants may have roles in disease pathogenesis. The biological significances urge studying the complexity of gene expression at the transcript level. This article updates the molecular basis of transcript variants in mammalian cells, including the formation mechanisms and potential roles in host biology. Gaining insight into the transcript variants will not only identify novel mechanisms of gene regulation but also unravel the role of the variants in health and disease.

## 1. Introduction

**Figure 1:**
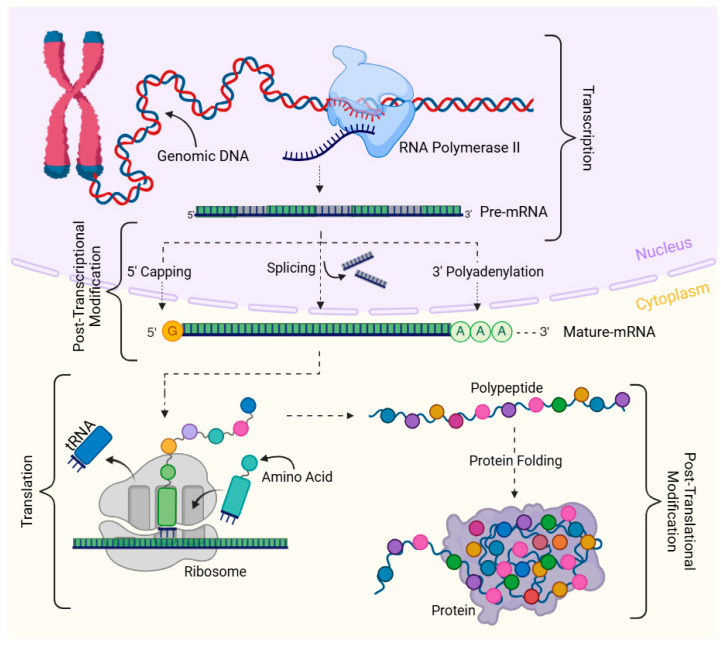
Overview of gene expression. Transcription incorporates various epigenetic and conformational changes in genomic DNA, which allows the copying of DNA into pre-RNA by RNA polymerase II in the cellular nucleus. Pre-mRNA undergoes post-transcriptional modifications, including splicing, 5′-capping (7-methylguanosine, (m7G) modification of the 5′ end of mRNA) and 3′-polyadenylation (addition of poly(A) tails to 3′ end of mRNA) to generate mature mRNA. Mature mRNA is exported to the cellular cytoplasm and translated to proteins.

Gene expression analyses remain the molecular basis of understanding cellular functions. The initial step in gene expression is the transcription of precursor mRNAs (pre-mRNA), which undergoes several processing steps to form mature mRNAs [1]. mRNAs are exported into the cytoplasm and translated into proteins [2]. Then, the proteins undergo post-translational modifications before targeting the sites of function [3]. The entire process—from transcription and post-transcriptional modifications to translation and post-translational modifications—ensures that the genetic codes are expressed accurately and contribute to cellular functions (Figure 1).

**Figure 2:**
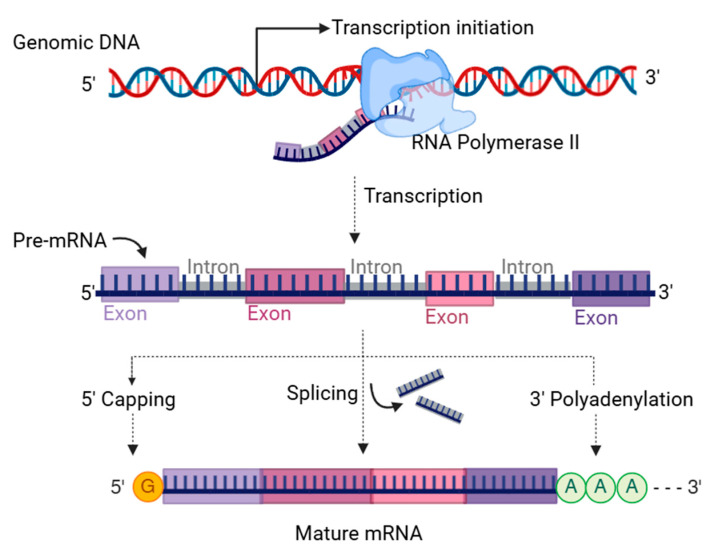
Transcription and post-transcriptional processing of pre-mRNAs. RNA polymerase II is involved in transcribing pre-mRNA from a gene. Introns are removed from the pre-mRNA by splicing. Capping at the 5′ end of pre-mRNA and polyadenylation at the 3′ end is necessary to form mature mRNAs. RNA binding proteins (RBPs) are essential for pre-mRNA processing and mRNA maturation.

Conventional gene expression analyses assume one gene expresses one mRNA that translates to a single protein [4]. However, the diversity of mRNA sequences and encoded proteins is much greater than the number of genes [5]. According to the Ensembl reference sequence (GRCh38.112), the human genome contains 47,873 genes, but the number of transcript variants is 237,285, about five times higher. Such an increased diversity in mRNAs and proteins occurs due to the expression of multiple mature mRNAs from a single gene, translating to various proteins [6,7]. The generation of transcript variants begins at the transcription step [2]. The enzyme RNA polymerase II catalyzes the synthesis of the pre-mRNA complementary to the genomic DNA template [8]. Transcription machinery, including RNA polymerase II, gains access to the target promoter site within the gene locus depending on epigenetic changes and the availability of transcription factors [9]. Initiation of gene expression from the alternative transcription start sites (TSSs) results in transcript variants at the 5′ end [10]. Moreover, alternative post-transcriptional processing, including alternative splicing of exons and alternative polyadenylation (APA) at the 3′ end, can contribute to forming multiple transcript variants [11,12] (Figure 2).

Despite their abundance, the significance of transcript variants is often underestimated, as traditional gene expression studies assume one gene expresses one mRNA [13]. This simplistic presentation of “one gene–one mRNA–one protein” is biologically inaccurate for most mammalian genes [14]. The concept does not reflect that most genes express multiple transcript variants, translating into different proteins or generating non-coding RNAs [6,7]. The three critical aspects of gene expression—the level of expressed genes, the regulation of expression, and the functions of the expressed genes—cannot be achieved without detecting and analyzing individual transcript variants. The same gene can express the transcript variants in different quantities, and the variants can be expressed from different promoters and translated into proteins with different functional domains [15]. Different transcript variants can be expressed in different tissues in variable quantities during various stages of development or aging [16]. Various proteins expressed by the transcript variants may also contribute to the development of pathological conditions [17]. Accordingly, the molecular basis of transcript variant formation, regulation, and function remains the mainstay for understanding gene expression in mammalian cells.

## 2. Formation of Transcript Variants

**Figure 3:**
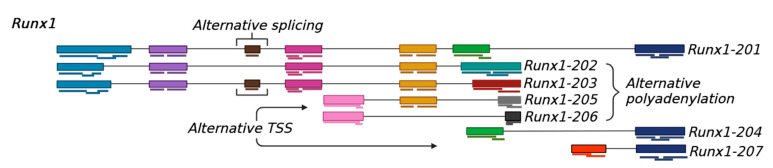
Expression of mRNA transcript variants. Schematic presentation depicts the generation of transcript variants of *Runx1* from a single gene locus. The transcript variants were expressed due to alternative transcription start sites (TSSs) (variants 204, 205, and 207), alternative splicing (variants 202, 203, 205, and 206), and alternative polyadenylation sites (variants 202).

mRNA transcript variants are various mature mRNAs expressed by a single gene [18]. As mentioned above, the transcript variants are generated due to alternative TSS, alternative splicing, and alternative polyadenylation. Thus, the transcript variants may differ in their 5′ end, internal exon composition, or 3′ end polyadenylation, leading to a diverse protein-coding capacity [19]. Here, we have shown a schematic presentation of the transcript variants expressed by the *Runx1* gene (Figure 3). Some of the *Runx1* transcript variants resulted from alternative TSSs (*Runx1-204*, *Runx1-205*, *Runx1-207*), others from alternative splicing (*Runx1-202*, *Runx1-203*, *Runx1-205*, *Runx1-206*), and APA (*Runx1-202*). It needs to be noted that several other mechanisms, like RNA editing, can generate transcript variants. In the following sections, we have described the mechanisms with simplified schematics.

### 2.1. Transcription from Alternative Start Sites

Epigenetic modifications are essential for accessing transcription machinery to the gene promoter, and RNA polymerase II catalyzes the pre-mRNA synthesis [8,20]. Transcription from alternative start sites allows gene expression from multiple locations on the same gene locus [21]. On average, a human gene may possess about four alternative TSSs [22]. A recent study has shown that 81% of mouse embryonic stem cell genes utilize alternative TSSs [23]. The variants are generated due to the initiation of transcription from different start sites within the 5′ end of a gene, either from the same or a different exon. Alternative start sites from different exons can lead to alternative splicing of the 5′ exons, generating identical transcripts [24]. These processes may result in mature mRNA variants with different non-coding 5′ untranslated regions (UTRs) or give rise to alternative start codons for protein translation [18]. When the transcript variants contain alternative start codons, the translated polypeptides may differ in the amino terminus [25].

**Figure 4:**
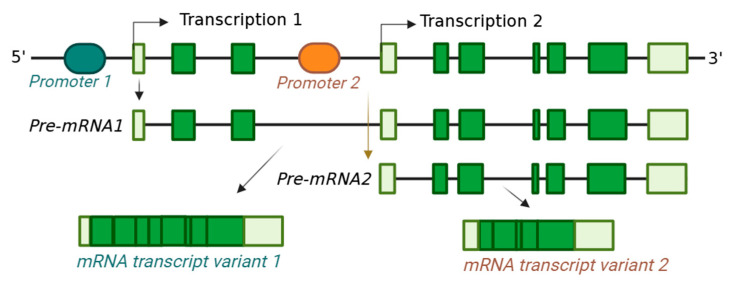
Transcription from alternative promoters of the same gene. Promotors are DNA sequences that allow RNA polymerase to bind and initiate transcription. Here, promotor 1 directed transcription at the start site, leading to a pre-mRNA and eventual mRNA transcript variant with a different 5′ end. Promotor 2 is an alternative promoter downstream of promoter 1, capable of directing transcription. The alternative promotors allow for the generation of distinct mRNA transcript variants. Cell-type-specific epigenetic changes or binding cell-type-specific transcription factors can activate specific promoters.

An alternative TSS may occur due to the alternative promoters within the 5′ end of a gene with different enhancers or repressors [26]. The selection of alternative promoters depends on the epigenetics of the proximal promoters in a cell type. However, the availability of cell-type-specific alternative transcription factor complexes also plays a crucial role in initiating transcription from alternative TSSs [27] (Figure 4). Different cell types may use alternative proximal promoters that alternative transcription regulators regulate. Accordingly, alternative TSSs are frequently found to generate tissue-specific mRNA variants [21].

### 2.2. Alternative Splicing of Pre-mRNA

**Figure 5:**
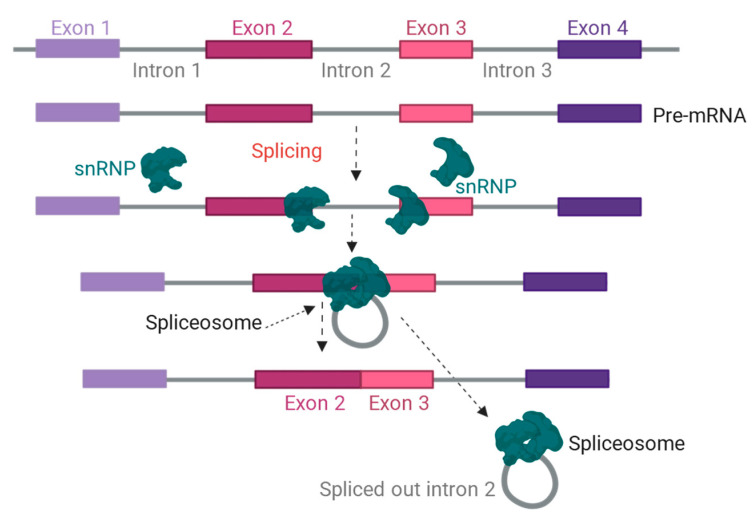
Overview of splicing mechanism. Small nuclear ribonucleoproteins (snRNPs) recognize and bind to the 5′ and 3′ splice sites. Other snRNPs join to form a complete spliceosome. The newly formed spliceosome brings in the exons for ligation and simultaneously removes the intron to complete the process.

Splicing is a cellular process for removing the introns from pre-mRNAs [28]. Precise splicing is essential for the generation of mature mRNAs. More than 97% of human genes have introns to be spliced for the maturation of mRNA [29]. During splicing, removing introns is concomitant with ligating the flanking exon sequences [30]. It consists of two stepwise transesterification reactions, which lead to a lariat formation followed by exon ligation and release of the intron lariat [31,32] (Figure 5). The transesterification reactions are catalyzed by the spliceosome, a macromolecular complex consisting of five small nuclear RNAs (snRNAs) and numerous RNA-binding proteins (RBPs). During the first transesterification, the 2′-OH group of the branch point adenosine reacts with the phosphate group at the 5′ splice site, cleaving the 5′ end of the exon and forming 2′, 3′ branched linkages [31]. In the second step, the 3′-OH group of the 5′ exon attacks the 3′ splice site, ligating the two exons and removing the intron lariat (Figure 5).

The spliceosome complex acts as the primary machinery for regulating RNA splicing and ensures the correct processing of pre-mRNA, which is essential for gene expression and protein synthesis [32,33,34]. There are two main kinds of spliceosomes: the major spliceosome and the minor spliceosome [35]. The major spliceosome is responsible for over 99% of pre-mRNA introns, while the minor spliceosome deals with a small subset of U12-type introns [36]. The major spliceosome consists of five snRNAs, U1, U2, U4, U5, and U6, and numerous RBPs, including snRNPs [37,38]. The minor spliceosome also contains five snRNAs, U11, U12, U4atac, U6atac, and U5, and unique sets of RBPs [38]. As the components of spliceosome complexes, many RBPs regulate the splicing mechanism [39,40]. These include serine/arginine (SR) proteins, heterogeneous nuclear ribonucleoproteins (hnRNPs), RBPs with RNA recognition motif (RRM), K homology (KH), and zinc finger (ZNF) domains [41,42]. Notable SR proteins include SRSF1 (ASF/SF2), SRSF2 (SC35), and SRSF3 (SRp20) [41,42]. SR proteins often act as splicing enhancers, facilitating exon inclusion. They also play a key role in regulating post-splicing events, including nuclear transport, translation, NMD, and miRNA biogenesis [43]. In contrast to the SR proteins, the hnRNPs typically act as splicing repressors, often binding to exonic splicing silencers (ESSs) [44,45]. Well-known hnRNPs are PTBP1, PTBP2, hnRNP A1, and hnRNP H. In addition to the SR proteins and hnRNPs, certain splicing factors exhibit tissue-specific activity. These include NOVA, MBNL, RBM20, nPTB, and RBFOX1 [46,47]. The tissue-specific regulators often work with the ubiquitous SR and hnRNP proteins to fine-tune splicing patterns in specific cell types [46,47].

**Figure 6:**
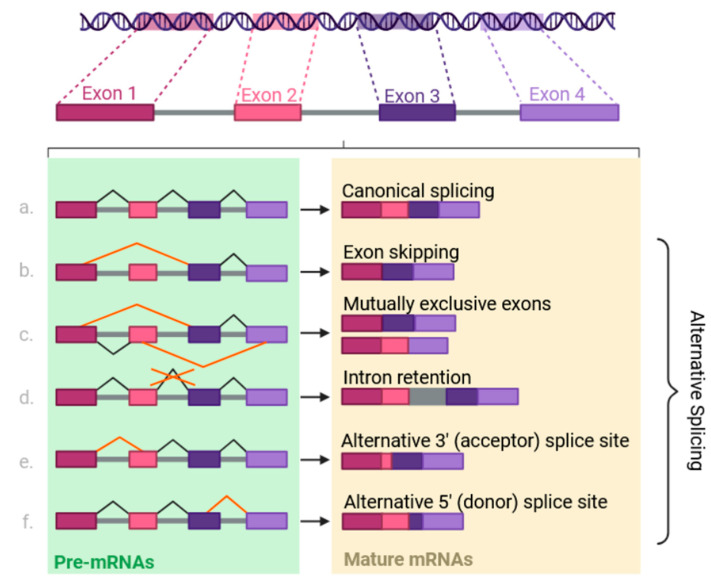
Types of RNA splicing in mammalian cells. Before becoming mature mRNAs, pre-mRNAs may undergo different types of alternative splicing. Canonical splicing (**a**) occurs when all introns are removed, and the exons remain in the same sequence. Exon skipping (**b**) excludes an exon from the splicing event, so the mRNA lacks that exon. Mutually exclusive exons (**c**) are a process in which only one internal exon is retained while all other neighboring internal exons are spliced. Intron retention (**d**) occurs when an intron stays within the mature mRNA sequence. Alternative 3′ splice sites (**e**), often called acceptor sites, occur when the 5′ end of a downstream exon is spliced, and the remaining 3′ end is added to the upstream exon to set a new 3′ boundary. Alternative 5′ splice sites (**f**), often called donor sites, occur when the 3′ end of an upstream exon is spliced out, and the remaining 5′ end is used as the new 5′ boundary for the downstream exon.

Alternative splicing allows the generation of multiple mRNA variants from a single pre-mRNA. Studies have suggested that alternative splicing is a conserved step in mRNA processing in all mammalian cells; 95% of human pre-mRNAs are alternatively spliced [48,49]. The alternative splicing mechanism involves exon skipping, mutually exclusive exons, intron retention, an alternative acceptor site, and an alternative donor site [50] (Figure 6). Exon skipping is the most common alternative splicing pattern, where a single exon is skipped during RNA splicing [50].

Alternative in-frame splicing results in mRNA variants that encode proteins with common amino acid sequences but different functional domains [15]. The proteins encoded by alternatively spliced transcripts may differ in binding properties, enzymatic activity, intracellular localization, stability, and post-translational modifications [50,51]. When alternative splicing changes the open reading frame or creates a premature stop codon, it fails to generate a functional protein [52]. Such transcript variants may undergo degradations like nonsense-mediated decay (NMD) [53]. Alternative splicing is not only crucial for physiological functions but also for pathological consequences.

### 2.3. Alternative Polyadenylation of mRNA

A post-transcriptional process adds adenine residues to the 3′ end of mRNA molecules, forming a poly(A) tail known as polyadenylation. Studies have reported that polyadenylation protects mRNA from degradation, increases stability, facilitates nuclear export, and enhances translation [54]. In eukaryotic cells, mRNA signals direct both polyadenylation and transcription termination [55]. It is suggested that both termination of transcription and polyadenylation are linked to the maturation of mRNAs [56]. Termination of pre-mRNA transcription can be directed by a poly(A) signal (PAS), but it is unclear how a PAS triggers RNA polymerase II to terminate [57]. Polyadenylation occurs both in the nucleus and cytoplasm of eukaryotic cells as well as in organelles like mitochondria [58,59].

**Figure 7:**
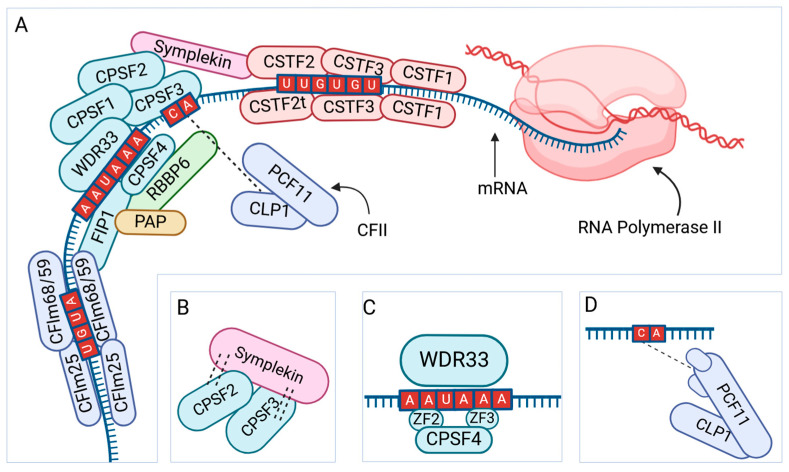
Overview of polyadenylation at the 3′ end of mRNA. The factors responsible for adding a poly(A) tail to the 3′ end of an mRNA (**A**). Notably, large CSTF and CFI complexes are composed of dimers and interact with U- and GU-rich sequences downstream, while FIP1 binds to U-rich elements upstream and can also interact with PAP cleavage. Symplekin binds with CPSF2 and CPSF3, forming a functional complex that can interact with other accessory proteins to complete pre-mRNA maturation (**B**). WDR33 can directly recognize the poly(A) signal, whereas CPSF4 binds using zinc fingers (**C**). PCF11 and CLP1 interact to form the CFII complex and aim for the cleavage site, mediated by two zinc fingers, located after a cytosine (**D**).

The first step of the polyadenylation process includes cleavage factor I (CFI), cleavage and polyadenylation specificity factors (CPSFs), and cleavage stimulation factors (CSFs), which facilitate the binding of the transcription complex to a polyadenylation signal sequence [60,61] (Figure 7). The transcriptional complex, consisting of RNA polymerase II and the factors mentioned above, initiates the cleavage of a short segment of pre-mRNA downstream to the polyadenylation site [62]. Then, a polyadenylation binding protein (PABPN1) binds to the 3′ end of the sequence and initiates the polyadenylation [62,63]. The process may add a tail of 50–250 adenosine nucleotides to the 3′ end of the pre-mRNA [63]. Altered expression of factors involved in transcription termination and polyadenylation may lead to APA and variation in the length of poly(A) tails [63].

**Figure 8:**
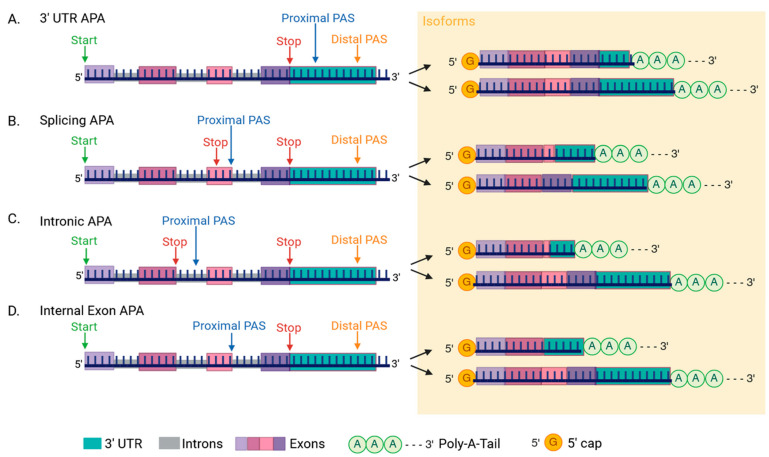
Types of mRNA polyadenylation in mammalian cells. (**A**) 3′ UTRs alternative polyadenylation (APA) occurs when multiple cleavage or polyadenylation sites (PASs) exist on the pre-mRNA transcript, creating transcript variants with different 3′ end lengths. (**B**) Splicing APA results after an alternative splicing event occurs with a PAS on a terminal exon, leading to exon skipping isoforms. (**C**) Intronic APA is found when a PAS is located within an intron. (**D**) Internal exon APA uses PAS on upstream exons to create isoforms missing a stop codon and 3′ UTR.

Alternative pre-mRNA cleavage and APA are widespread mechanisms to generate mRNA transcript variants with diverse 3′ ends. Multiple polyadenylation sites in a pre-mRNA sequence can lead to APA [64]. APA can also be associated with different lengths of the poly(A) tail [65]. APAs are often tissue-specific and may play a key role in cell proliferation and differentiation [60]. There are two types of APA, tandem 3′ UTR-APA and upstream APA (UR-APA) [66] (Figure 8). The APA subtypes diversify protein expression or inhibit gene expression detected during cell differentiation [67,68,69]. The 3′ UTR-APA is more common; it changes the lengths of 3′ UTRs and impacts mRNA stability, nuclear transport, and translation [61]. In contrast, the UR-APA can change the 3′ coding sequences by alternative splicing or intron retention [69,70]. Several factors are known to be involved in the cleavage of the 3′ end of mRNA and APA.

### 2.4. RNA Editing

The formation of transcript variants due to RNA editing is infamous for Trypanosoma pathogenesis, which helps the parasites to invade the host’s immune system [71]. However, RNA editing also occurs in higher eukaryotes, including mammals [72]. RNA editing leads to site-specific changes in the nucleotide sequence of mRNAs through two distinct mechanisms: substitution and insertion/deletion [73].

In substitution editing, individual nucleotide bases are chemically altered and catalyzed by enzymes, such as adenosine deaminases or cytidine deaminases [74]. Both adenosine deaminases and cytidine deaminases remove an amino group that alters and replaces a nucleotide base in RNA. Adenosine deaminases acting on RNAs (ADARs) convert adenine (A) to inosine (I), known as A-to-I in RNA editing [75,76]. Five double-stranded RNA-specific ADAR enzymes are encoded by three *ADAR* genes in mammalian cells, namely ADAR1(p110), ADAR1(p150), ADAR2a, ADAR2b, and ADAR3 [77,78]. The ADAR enzymes differ in adenosine deaminase activity and target their substrate preferences [79,80,81]. Cytidine deaminases convert cytosine (C) to uracil (U), which is another key mechanism of RNA editing [75]. The APOBEC proteins are well-known cytidine deaminases expressed in human cells [75]. Both A-to-I and C-to-U conversions alter the codon sequence to code for different amino acids, which can change the protein properties [82]. RNA editing is essential for regulating gene expression and correcting mutations, neuronal functions, and neurodegenerative diseases [83]. Recent studies show that ADAR-based mRNA editing has the potential for developing therapeutic strategies [84].

In insertion/deletion editing, nucleotides are integrated into the RNA sequence or are removed [85]. Complementary guide RNA molecules mediate these reactions, pair them with the RNA bases, and serve as a template for adding or removing targeted nucleotides [86]. Guide RNA molecules pair with the RNA bases and serve as a template for adding or removing targeted nucleotides. Insertion or deletion into pre-mRNA sequences can impact RNA splicing, stability of the mRNA, and coding of proteins [87]. Thus, mRNA editing increases the number of transcript variants and diversity in protein sequences without having a mutation in the genomic DNA sequences.

### 2.5. Genetic Mutations

Genetic mutations can also generate mRNA transcript variants [88,89]. Mutations can change single nucleotides, like missense or nonsense mutations or multi-nucleotides, like insertion or deletion mutations [88]. Mutation in the promoter region, particularly in the enhancer or repressor elements, can result in alternative TSSs [90]. While a frameshift mutation can result in mRNA expressing nonfunctional or harmful proteins, nonsense mutations can introduce premature stop codons, rendering such transcript variants to undergo decay processes [91,92]. A gene mutation can also affect the cis-regulatory elements of pre-mRNA, like splice donor or acceptor sites, resulting in alternative splicing and generating transcript variants [39]. Mutations can also lead to APA and generate mRNA transcript variants at the 3′ end of mRNA [7]. It has been reported that *APC* gene mutations can dysregulate APA in cancers [93].

## 3. Regulation of Transcript Variant Formation

### 3.1. Regulatory Steps

Transcription and post-transcriptional modifications of pre-mRNA are intricately linked events, co-occurring on the transcribing genes [94]. Thus, pre-mRNA processing is also influenced by transcription machinery, epigenetic regulators, and transcription factors that primarily control transcription. Epigenetic and transcriptomic regulators control transcription from alternative TSSs, resulting in transcript variation at the 5′ end of mRNAs [21,95]. Therefore, alternative promoters can be activated in a tissue or developmental stage-specific manner [96,97,98]. Recent studies have shown that alternative TSSs can regulate downstream pre-mRNA processing and mRNA transcript variant selection [21,95].

In addition, the generation of transcript variants is regulated at the level of splicing and APA at the 3′ end of mRNAs. The regulators of epitranscriptomic dictate alternative splicing and APA [99]. Splicing factors and cis-regulatory elements regulate pre-mRNA splicing; thus, alternative splicing can be tissue-specific, developmental stage-specific, and linked to disease conditions [100,101,102].

### 3.2. Regulation by RNA Modifications

**Figure 9:**
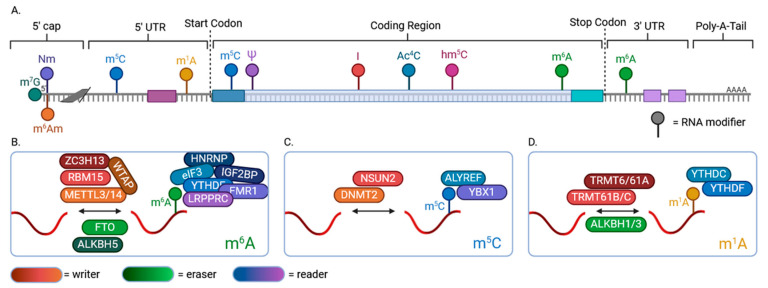
Chemical modification of mRNA and the RNA modifiers. (**A**) Schematic presentation of RNA modifications on an mRNA strand. (**B**) m^6^A is located within the 3′ UTRs, near stop codons, and it is regulated by many writers, readers, and erasers, such as the notable METTL3/14, YTH proteins, and obesity-associated proteins of FTO and ALKBH5. (**C**) m^5^C is catalyzed by the NSUN domain proteins and DNA methyltransferase, has two identified readers in ALYREF and YBX1, and occurs within the 5′ UTRs near the start codon or the coding and non-coding 3′ UTRs. (**D**) m^1^A can be found primarily within the 5′ UTRs at the beginning of transcripts or within coding sequences, and it is regulated by TRMTs, YTHDC/F, and ALKBH1/3 demethylases.

Besides the 5′-capping (7-methylguanosine; m7G), more than 300 distinct chemical modifications occur in RNA bases, which defines the cellular epitranscriptome [103]. RNA modifications are more common than DNA or histone modifications, suggesting that cellular epitranscriptome plays a more complex role in gene regulation downstream to the epigenome [104,105]. Despite the vast number of chemical modifications of RNA bases, the commonly studied ones are m7G, N6-methyladenosine (m6A), adenosine to inosine (A-to-I), N6,2′-O-dimethyladenisine (m6Am), 5-methylcytosine (m5C), N1-methyladenosine (m1A), N4-acetylcytosine (Ac4C), and pseudouridine (ψ) due to occurrence and functions [11,106] (Figure 9A). Most RNA modifications are reversible and regulated by abundant writers, readers, and erasers.

It is reported that m6A is catalyzed by METTL3/METTL14 and regulated by the YTH proteins and obesity-associated proteins FTO and ALKBH5 (Figure 9B). Other RNA modification like m^5^C is catalyzed by the NSUN proteins and DNA methyltransferase and regulated by the readers, ALYREF and YBX1 (Figure 9C), while less frequent m^1^A uses TRMTs as mRNA writers, YTHDC/F as readers, and ALKBH1/3 demethylates (Figure 9D). m6A modification involves the target sequence motif of RRACH (R = G/A; H = A/C/U), and it predominantly occurs in the last exons within 3′ UTRs near stop codons or the coding sequences of mRNAs, especially in long internal exons [60,107,108,109,110,111]. In contrast, m5C modifications are found within the 5′ UTRs, near the start codons of mRNAs, but they can also occur within the coding sequences, introns, or 3′ UTRs [60,107,108,109,110]. Similarly, m1A modifications are enriched in the 5′ UTRs of mRNAs at the beginning of the transcripts. However, it can also be found within the coding sequences [112,113]. Some RNA modifications also convert RNA bases like A-to-I and C-to-U, as discussed in the RNA editing section (Section 2.4).

RNA modifications play crucial roles in RNA biology, from synthesis to the function of different RNAs [11,106,109,114]. The chemical changes to RNA bases alter their structure and impact interactions of RNA molecules with RBPs [115,116]. Those are critical regulators of pre-mRNA processing, including splicing, polyadenylation, nuclear export, transcript stability, and translation regulation [116,117,118]. RNA modifications are essential for the accuracy and efficiency of RNA splicing, and changes in RNA modifications can lead to alternative splicing [119,120]. The modifications can also impact polyadenylation by affecting the interaction with the respective RBPs. It is well-established that m7G forms the 5′ cap structure of mRNAs, which is conserved in all mature mRNAs and essential for the initiation of translation [111,114]. The other novel modification, m6A, is abundant and conserved in eukaryotic mRNAs [109,114]. It is found in ~30% of all mammalian mRNAs, with three to five modification sites occurring per transcript [109]. m6A is important for the abovementioned functions of RNA modifications, as well as signal transduction and transcriptional regulation [109,121,122]. As the most prevalent internal mRNA modification, m6A modifications are the most important regulator of pre-mRNA processing and formation of transcript variants [108,121,123]. Other modifications, like m5C, also play a crucial role in RNA metabolism; those are found in both mRNA and tRNA and regulate tRNA functions during translation [115].

Changes in RNA modifications can impact transcription, pre-mRNA processing, and polyadenylation, which can lead to the generation of alternative mRNA transcript variants [116,117,118]. These modifications are also crucial for cell-type-specific transcript variants, essential for maintaining cellular identity and function. The interplay between RNA modifications and the diversity of transcript variants is crucial for maintaining cellular homeostasis and responding to environmental changes [106,115]. By modulating the stability, processing, or translation, RNA modifications selectively promote or inhibit the expression of specific transcript variants, influencing cellular functions and phenotypes [124]. Transcript variants allow the cells to fine-tune gene expression in response to various stimuli, improving their adaptability. RNA modifications can change under stressful or disease conditions, giving rise to condition-specific transcript variants [125]. This response facilitates cellular adaptability to environmental changes or pathological conditions [126,127].

Despite the crucial role, the detection and mapping of RNA modification remain relatively tricky. RNA modifications can be quantified using liquid chromatography-mass spectrometry (LC-MS), but specific modifications can be mapped only using transcriptome-wide high-throughput sequencing. Antibody-based immunoprecipitation of the modified bases in RNA transcripts can be used to prepare libraries for high-throughput sequencing. Recent advancements in methylated RNA immunoprecipitation sequencing (MeRIP-Seq) have enhanced the detection and mapping of specific modifications within the whole transcriptome [128,129]. In addition, the Oxford nanopore technology-based direct RNA sequencing can also identify RNA modifications by analyzing changes in signals as RNA passes through the nanopores [130]. Future development detection and mapping of RNA modifications is crucial for a better understanding of epitranscriptomic regulation of gene expression and the generation of mRNA transcript variants [131].

## 4. Role of the mRNA Transcript Variants

### 4.1. Coding Proteins with Different Domains

Despite the identical genomic code, the mRNA transcript variants may encode distinct proteins with diverse functions [132]. Each transcript variant may encode proteins with different combinations of polypeptide sequences. Alternative splicing in the same open reading frame of the original mRNA generates transcript variants that encode proteins of identical amino acid sequences but truncated at either end or the internal region [132,133]. Alternative TSSs give rise to transcript variants that differ in the 5′ end and may possess alternative start codons, resulting in different leader peptides targeted to different organelles [134]. Such distinct proteins translated from the transcript variants expressed by the same gene may have different functions [19]. However, alternative TSSs or alternative splicing may also result in transcript variants failing to code meaningful peptides [135]. APA, particularly UP-APA, can lead to transcript variants that encode proteins with a diverse carboxy terminus. Moreover, diverse RNA modifications may quantitively alter the mRNA transcript variants and their capacity for protein-coding [19,136].

### 4.2. Non-Coding RNAs

Non-coding RNAs (ncRNAs) do not code for proteins but play a crucial regulatory role in gene expression [137]. ncRNAs can form due to post-transcriptional processing of pre-mRNA [138]. Non-coding RNAs mainly arise through alternative TSSs, alternative splicing, or APA, resulting in the loss of start or stop codons [139]. The loss of other crucial segments of pre-mRNA may also result in transcript variants that may not encode a functional protein and, therefore, become ncRNAs [140]. There are two types of ncRNAs: long non-coding RNAs (lncRNAs) or small ncRNAs (like microRNAs) [141]. lncRNAs do not code for proteins but regulate other cellular functions, such as gene expression and the epigenetic state of a gene [142]. Small ncRNAs can significantly impact transcriptional silencing of a gene, mRNA stability, and translation of an mRNA [143].

### 4.3. Decay of mRNAs

The decay of mRNAs is a mechanism that regulates the level of gene expression [144]. In this process, mRNAs are degraded after their functional lifespan, maintaining enough mRNA within the cells [145]. The decay of mRNAs with inaccurate sequences avoids abnormal protein production [146]. Among the pathways of mRNA decay, the most common is the general cytoplasmic mRNA turnover [147]. In the cytoplasm, the poly(A) tail is shortened by deadenylases, which open the circular loop and expose the 5′ end for degradation by exonucleases like XRN1 [148]. Alternatively, mRNA degradation can occur from the 3′ end through an exosome complex [149].

In addition to these general pathways, several specialized pathways are quality control measures that ensure proper protein synthesis. These include NMD that target mRNAs with premature stop codons [92]. Premature stop codons can lead to truncated proteins, potentially harmful to cells. During NMD, specialized factors, such as up-frameshift (UPF) proteins, form the main surveillance machine and recognize defective or nonfunctional transcripts [91]. Exon junction complexes (EJCs) also help distinguish premature stop codons from the other functional codons [150]. Then, those transcripts are guided to the standard decay machinery, a suppressor with a morphogenetic effect on genitalia (SMG) proteins, which upregulate UPFs and recruit decaying enzymes [151]. Another mRNA decaying mechanism is the nonstop decay (NSD) that targets mRNAs without a stop codon [152]. Moreover, the non-go decay (NGD) pathway degrades mRNAs with damaged codons or structural irregularities [153]. The pathways of mRNA degradation remove the mRNAs that may translate to defective or harmful peptides.

## 5. Transcript Variants in Physiology and Pathological Conditions

Transcript variants can encode proteins with diverse functional domains or form noncoding regulatory RNAs, modifying cellular functions [138]. Identifying the transcript variants makes it possible to understand how genes are expressed in physiological conditions and how they contribute to diseases. Thus, studying the mRNA transcript variants is essential for advancing our understanding of gene expression and its implications for health and diseases. In the following sections, we have discussed the importance of studying transcript variants in normal physiology, disease pathology and the potential for translational research.

### 5.1. Transcript Variants in Physiological Conditions

Post-transcriptional pre-mRNA processing and the generation of mature mRNA transcripts regulate diverse biological processes essential for normal development and aging [154]. In the human brain, 1174 exons are differentially expressed with aging. These changes are associated with decreased alternative mRNA splicing in human blood and brain and in rodent brain, skin, muscle, bone, thymus, spleen, and adipose tissues [155,156]. The alternative splicing mechanism may result from changes in RNA polymerase II elongation, which increases with age across mice, rats, humans, nematodes, and fruit flies [155,156]. These changes correlate with increased spliced transcripts and an increased formation of circular RNAs. Moreover, accelerated transcription and splicing increased the changes of erroneous splicing events, which is detrimental to cellular functions and could result in cell death [157]. Nevertheless, whether these changes are drivers of aging or consequences of age-related processes remains unclear.

Since aging involves cross-communication and rapid changes in many physiological systems, it can be challenging to follow the process on a molecular scale. However, there is an indication that *Cdkn1a* transcript variant 2, which encodes the *p21Cip1/Waf1* protein, appears to be a specific marker of aging and cellular senescence [158]. In aging mice, *Cdkn1a* transcript variant 2 was selectively elevated in the liver, adipose, kidney, heart, and lung [158]. These findings suggest that future studies at the transcript level may discover more specific markers and cellular functions during development and aging.

### 5.2. Transcript Variants in Pathological Conditions

Altered pre-mRNA processing and the formation of mRNA transcript variants have been shown to play a critical role in disease development [159]. Transcript variants may contribute to pathogenesis by encoding proteins with altered gain-of-function or loss-of-function properties [160]. An abnormal variant may also encode proteins that localize or accumulate in unexpected cellular compartments [161]. Studies have shown that expression of alternative transcript variants can be associated with neurodegeneration, cardiovascular diseases, autoimmunity, and cancers [39].

The canonical form of α-synuclein remains closely tied to the pathologic protein aggregation and neuronal damage causing sporadic Parkinson’s disease [162]. An alternative variant of α-synuclein, which shows a lower expression level, displays less protein aggregation than the full-length α-synuclein [163]. In retinitis pigmentosa, mutations in core spliceosome components such as PRPF31 and PRPF8 disrupt photoreceptor-specific splicing, leading to retinal degeneration [164]. RNA gain-of-function mechanisms are implicated in diseases like myotonic dystrophy (DM1 and DM2), where expanded CUG or CCUG repeats sequester splicing regulators like MBNL1, causing widespread mis-splicing [165]. The altered transcript variants of the CACNA1C gene can result in different types of Timothy syndromes, depending on which one is affected [166]. Additionally, errors in RNA editing mediated by ADAR enzymes can lead to improper protein function and neurological disorders [167]. In neurodegenerative diseases like amyotrophic lateral sclerosis (ALS), mutations in RNA-binding proteins such as TDP-43 and FUS impair splicing and RNA transport, causing widespread neuronal dysfunction [167,168]. Similarly, fragile X-associated tremor ataxia syndrome (FXTAS) involves CGG repeat expansions in the FMR1 gene, sequestering RNA-binding proteins and disrupting translation regulation [165]. Spinal muscular atrophy (SMA) is another example where mutations in the SMN1/SMN2 genes reduce the assembly of U snRNPs, impairing splicing and motor neuron survival [169].

In addition to neurodegenerative diseases, the formation of mRNA transcript variants has been reported in cardiovascular and metabolic diseases. Due to alternative splicing, multiple transcript variants are produced from the *VEGFA* gene by exon-skipping [170]. The functional domains of VEGF are essential in cardiac health, and a lack of exons 6 and 7 exhibit angiogenic abnormalities and ischemic cardiomyopathy [170]. However, alternative splicing of specific exons allows for the pro-angiogenic effects of VEGF [170]. The insulin receptor gene expresses two alternative splice variants that differ in quantity [171]. Discrepancies in expression profiles, irregular ratios, and dysregulation of both insulin receptor variants are associated with diabetes [171].

Cancer is also heavily influenced by RNA processing defects. Aberrant splicing caused by deregulated splicing factors promotes tumor progression in cancers like liver, lung, and breast cancer. For instance, alternative splicing can activate oncogenes or inactivate tumor suppressors [164,172]. Nonetheless, future studies will determine how specific mRNA transcript variants regulate disease progression.

### 5.3. Translational Relevance of Studying mRNA Transcript Variants

Pathological conditions are associated with differential expressions of mRNA transcript variants, which have the potential for diverse protein expressions. Such disease-specific variants can be considered novel diagnostic or prognostic markers and potential therapeutic targets [13]. Transcript variant profiling can also help to understand an individual’s unique genetic makeup. Thus, analyses of transcript variants expressed in a patient may help predict drug response and disease prognosis, improving personalized medicine [173]. The correct deposition of RNA modifications, the primary regulator of transcript variant formation, has been related to disease pathogenesis [116,154]. m6A and its demethylase ALKBH5 are associated with cancer development and progression, making it a potential target for therapeutic interventions [128,174,175].

mRNA transcript variants have another significant clinical implication regarding the therapeutic use of interfering RNA (iRNA) and antisense oligonucleotide (ASO) [176]. These promising therapeutic approaches can treat various diseases from refractory to conventional treatment strategies. ASOs and RNAi therapies can also target specific genetic mutations and can be used to develop personalized treatments and precision medicine [177]. However, before using ASOs or RNAi, it must be considered that some mRNA transcript variants may not possess ASO or RNAi target sequences due to alternative TSSs, splicing, or APA [13,178]. This issue limits the use of ASO or RNAi therapy, in addition to their existing challenges with off-target effects.

## 6. Conclusions

While analyzing the levels of gene expression or regulation of gene expression, researchers often assume that a gene expresses a single mRNA that is translated into a protein. However, the biology of gene expression is complex, where multiple mRNA transcript variants are expressed from a gene, which can vary in the 5′ end, 3′ end, or internal coding sequences. Thus, a single gene can express multiple proteins with diverse functional domains. Moreover, the transcript variants may be expressed in different quantities in different cell types or within the same cell type during different physiological or pathological conditions. The transcript variants can play a crucial role in cell differentiation, developmental organogenesis, and aging processes. Recent studies suggest that the expression of alternative transcript variants can be linked to disease pathogenesis. In this article, we have discussed the transcriptional and post-transcriptional processes that can result in the expression of mRNA transcript variants. However, tRNA, other small RNAs, and rRNAs, which play an essential role in pre-mRNA processing and translation, also form transcript variants. However, it remains unclear how these transcript variants impact the generation and function of mRNA transcript variants.
